# Subtle Deregulation of the Wnt‐Signaling Pathway Through Loss of Apc2 Reduces the Fitness of Intestinal Stem Cells

**DOI:** 10.1002/stem.2712

**Published:** 2017-10-13

**Authors:** Madeleine A. Young, Carl S. Daly, Elaine Taylor, Rhiannon James, Alan Richard Clarke, Karen Ruth Reed

**Affiliations:** ^1^ Cardiff School of Biosciences European Cancer Stem Cell Research Institute Cardiff Wales United Kingdom; ^2^ Department of Health and Applied Science University of the West of England Bristol United Kingdom

**Keywords:** Stem cells, Cell signaling, Animal models, Gene expression, Differentiation

## Abstract

The importance of the Wnt‐signaling pathway on the regulation and maintenance of the intestinal stem cell (ISC) population is well recognized. However, our current knowledge base is founded on models using systems of gross deregulation of the Wnt‐signaling pathway. Given the importance of this signaling pathway on intestinal homeostasis, there is a need to explore the role of more subtle alterations in Wnt‐signaling levels within this tissue. Herein, we have used a model of *Apc2* loss to meet this aim. *Apc2* is a homolog of *Apc* which can also form a destruction complex capable of binding β‐catenin, albeit less efficiently than *Apc*. We show that systemic loss of Apc2 results in an increase in the number of cells displaying nuclear β‐catenin at the base of the intestinal crypt. This subsequently impacts the expression levels of several ISC markers and the fitness of ISCs as assessed by organoid formation efficiency. This work provides the first evidence that the function and fitness of ISCs can be altered by even minor misregulation of the Wnt‐signaling pathway. Our data highlights the importance of correct maintenance of this crucial signaling pathway in the maintenance and function of the ISC population. Stem Cells *2018;36:114–122*

**Significance Statement**

To address the importance of subtle Wnt‐signaling deregulation on intestinal stem cell (ISC) function, this population of cells was analyzed within an Apc2^−/−^ mouse model. It was shown that the ISC population is highly sensitive to changes in Wnt‐signaling levels even when such changes are below the threshold that affects the maintenance of intestinal homeostasis.

## Introduction

The rapid turn‐over of cells within the intestinal epithelium is sustained by the presence of intestinal stem cells (ISCs) at the base of the intestinal crypt. The ISC population is tightly regulated in order to maintain intestinal homeostasis, and much of this regulation is controlled by the canonical Wnt‐signaling pathway. The canonical Wnt‐signaling pathway functions through β‐catenin, which builds up within the nucleus when a Wnt ligand is bound. Upon entering the nucleus, β‐catenin acts as a transcriptional co‐activator for Transcription Factor 4 (Tcf4), resulting in expression of a range of genes. In the absence of a Wnt ligand, a β‐catenin destruction complex consisting of GSK3β, Axin and adenomatous polyposis coli (APC), targets β‐catenin for phosphorylation, thereby depleting intracellular levels and preventing nuclear accumulation 1.

The role of Wnt signaling in the maintenance of the ISC compartment has been well described. Previous studies have shown that deficiency of Tcf4 (which effectively switches off Wnt signaling) results in a lack of ISCs during development 2. Alternatively, loss of the Wnt‐repressor Apc (which results in increased accumulation of nuclear β‐catenin and consequent activation of the Wnt‐signaling pathway) was shown to result in a gross expansion of undifferentiated cell types within the small intestine 3.

A homolog of *Apc*, *Apc2*, was first identified in drosophila and the structural similarities between the two proteins have led to the hypothesis that there may be some functional redundancy in the system 4. This is supported by the fact that Apc2 is evolutionarily conserved throughout a range of species from drosophila, mice, and humans 4.

Of the structural similarities between APC2 and APC, the presence of several 20‐amino acid repeat motifs is the most striking. It is these regions within Apc and Apc2 which are capable of binding β‐catenin within the destruction complex and thereby regulate Wnt signaling 5. However, Apc contains a 15‐amino acid repeat motif, lacking in Apc2, which binds β‐catenin at a higher affinity 6. The drosophila homolog of APC2 has been shown to have Wnt‐regulating capabilities, through its ability to rescue mis‐expression of Wnt target‐genes following loss of Apc 7. APC2 has also been shown to be capable of depleting the intracellular levels of β‐catenin in a human colorectal cancer (CRC) cell line through the formation of an APC‐independent β‐catenin destruction complex 8. However, in this context, APC2 depleted intracellular β‐catenin less efficiently than APC. In contrast, in APC deficient CRC cells, transient expression of APC2 resulted in a reduced readout in a TCF‐Topflash assay equivalent to that seen when APC is transiently expressed 4.

Activation of aberant Wnt signaling within the intestine is recognized to drive the development of CRC. Indeed, mutations within *APC* are common in this disease, being seen in more than 80% of all cases 9, whereas mutations in *APC2* are rarer (22 out of 619, <4%, cBioPortal 10, 11, 12). Despite this rarity, loss of gene function is also possible through other mechanisms. One such mechanism, epigenetic regulation of gene expression through the process of DNA methylation, is a common means for controlling gene expression. Hypermethylation of the promoter regions of tumor suppressor genes is commonly observed in a number of cancer types, and is a mechanism by which many non‐commonly mutated genes are downregulated 13, 14, 15. *APC2* has been shown to be hypermethylated in more than 90% of CRC tumors, indicating that downregulation of expression of *APC2* is likely to be an important step in colorectal tumorigenesis 16.

It is widely believed that the ISC is not only crucial in the homeostatic maintenance of normal intestinal physiology, but this important population of cells also plays a critical role in intestinal malfunctioning, such as the development of CRC. Thus, understanding the factors that regulate the number and function of ISCs is vitally important.

Although the effect of gross Wnt‐misregulation on the ISC population has been widely studied, less is known about the effect of more subtle Wnt‐regulation on this cell compartment. In order to assess the consequences of subtle deregulation of the Wnt pathway on the ISC compartment, here we have used a mouse model carrying a mutation for constitutive loss of *Apc2*.

## Materials and Methods

### Animal Models

Unlike knockout of its homolog Apc 17, a constitutive knockout of Apc2 does not result in embryonic lethality. The *Apc2^−/−^* mouse was produced by Professor Hans Clevers’ laboratory and gifted to us for analysis of the intestinal phenotype. The *Apc2^−/−^* mouse was produced by the insertion of a neomycin cassette including a stop codon into the open reading frame of exon 14, resulting in the production of an interrupted protein, lacking several β‐catenin, and axin binding sites 18, 19, 20.

### Quantitative Reverse Transcriptase Polymerase Chain Reaction (PCR) Analysis

Upon intestinal dissection, the first 10 cm of the proximal small intestine was taken for epithelial cell extraction. This was performed using the method outlined by Weiser, to produce an epithelial cell enriched population 21, which was then divided into three samples and stored at −80°C until required. Total RNA was extracted using the Trizol method. Complimentary DNA was transcribed from 5 µg of RNA using Superscript III (Invitrogen UK) and random hexamer primers (Promega).

Relative quantification was carried out using either the Fast Sybr Green master mix system (Promega), or the Taqman Universal mastermix system (Applied Biosystems) for genes with lower levels of expression. All samples were run in triplicate on the StepOne Plus PCR machine and the threshold cycle values of each gene were normalized to expression of β‐actin. Primer details can be found in the Supporting Information Methods.

### Sample Preparation and Immunohistochemistry

After dissection of the 10 cm of small intestine required for epithelial cell extraction, the remaining small intestine and colon was flushed with cold water, opened longitudinally and rolled from the proximal to the distal end. The resulting “swiss roll” was secured with a pin and placed in 10% formalin for 24 hours at 4°C, and processed into paraffin blocks. Five micrometer sections were cut and rehydrated into water. The sections were then either stained with haematoxylin and eosin for counting to enable histological analysis of apoptotic and mitotic bodies, or immunohistochemistry (IHC) was performed for β‐catenin expression. IHC was performed using Mouse Envision+ kit according to manufacturer's instructions with mouse mAB anti‐β‐catenin antibody 1:200 (BD, U.K.).

Apoptotic bodies were identified by their distinctive morphology of detachment from neighbors combined with highly condensed chromatin using H&E sections, as described by Potten et al. 22. Apoptotic index was calculated by average number of apoptotic bodies per half crypt divided by the average number of cells per half crypt for each individual mouse, counting >50 crypts per mouse. For cell position analysis, position 0 was counted at the bottom of a half crypt, and the cells were numbered sequentially up to the isthmus which marks the top of the crypt.

### In Situ Hybridization

In situ hyrbidization for Olfm4 was performed on formalin‐fixed paraffin‐embedded tissue prepared as for IHC. Protocol used was as outlined by Parry et al. 23.

### Intestinal Organoid Culture

Intestinal organoid culture was performed from small intestinal crypt preparations as previously described 24. Organoid formation efficiency was calculated as number of grown organoids (over 150 µm in length) after 10 days in culture as a percentage of the number of crypts seeded at day 0, as measured using GelCount software (see Supporting Information Methods).

At day 10, organoids were triturated to single crypts and re‐seeded as before. Self‐renewal was measured as the number of organoids which had grown after 10 days post passage as a percentage of the number which were seeded.

### Mitochondrial Activity Assay

Prestoblue (Invitrogen) was incubated 1:10 in the organoid media (and negative controls) for 2 hours at 37°C, then removed and placed into a 96 well flat‐bottomed plate for reading levels of fluorescence.

### Fluorescence‐activated cell sorting (FACs) of ISC

Intestinal epithelia of both *Apc2^+/+^* and *Apc2^−/−^* mice carrying the *Lgr5Cre‐GFP* 25, were disassociated to single cell level as described by Sato et al. 24. Cells were then incubated with 1:1,000 DAPI (4′,6‐diamidino‐2‐phenylindole) and sorted in a FACs medium of 1% Bovine Serum Albumin (BSA) and 0.1 mg/ml DNAseI in Dulbecco's Modified Eagle's medium (DMEM) F12 for expression of Green Fluorescent Protein (GFP) from the live cell population.

### Statistics

All data analysis was carried out using SPSS statistical software (IBM). A two‐tailed Student's *t* test was performed for all normally distributed data, while a nonparametric Mann‐Whitney test was used for all other data sets.

## Results

### Apc2 Regulates Wnt Signaling Within the Murine Small Intestine

**Figure 1:**
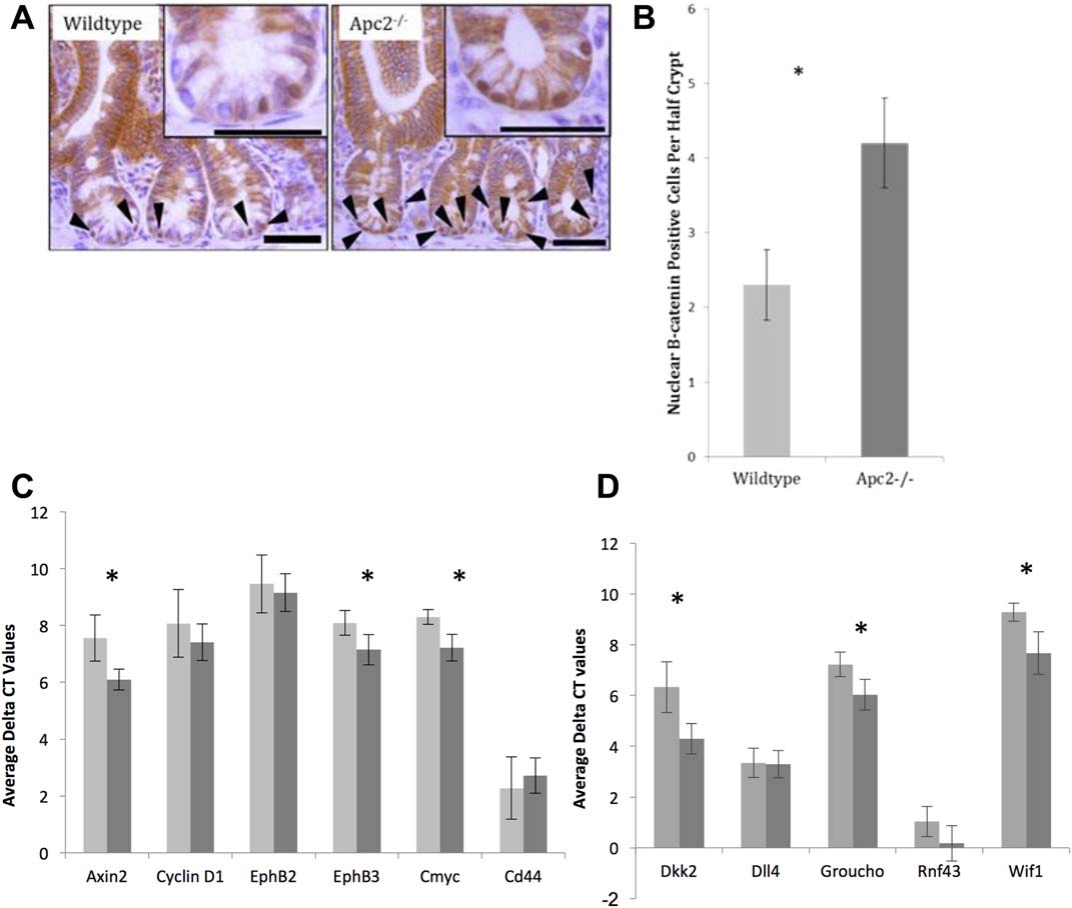
Apc2 deficiency subtly regulates Wnt signaling. **(A):** Immunohistochemistry for β‐catenin shows an increase in nuclear localization of β‐catenin. **(B):** The increase in nuclear β‐catenin is shown to be statistically significant *p* < .03 (*n* = 4). **(C):** Average delta CT values of Wnt target genes show a significant increase in expression of Axin2, EphB3, and Cmyc in intestinal epithelial tissue as a result of loss of Apc2 (*n* = 6). Light gray bars represent wildtype tissue, dark gray bars represent Apc2^−/−^ tissue. Asterisk denotes statistical significance. **(D):** Average delta CT values of Wnt repressor genes show a significant increase in expression of Dkk2, Groucho, and Wif1 in intestinal epithelial tissue as a result of loss of Apc2 (*n* = 5). Light gray bars represent wildtype tissue, dark gray bars represent Apc2^−/−^ tissue. Asterisk denotes statistical significance. Abbreviation: CT, cycle threshold.

In order to assess the impact of Apc2 loss on Wnt signaling transduction within the intestine, immunohistochemical analysis of β‐catenin localization was performed on intestines from *Apc2^−/−^* and *Apc2^+/+^* litermates. This analysis uncovered a significant increase in the number of cells possessing nuclear β‐catenin in an Apc2 deficient environment (Fig. 1A, 1B). The increase in nuclear β‐catenin as a result of *Apc2* deficiency is more subtle than that observed as a result of *Apc* loss, and is restricted to the base of the crypts 3. However, this finding is consistent with the notion that Apc2 contributes to the formation of a β‐catenin destruction complex within the normal intestinal crypt cells. It has been shown that increased Wnt‐signaling as a result of increase nuclear β‐catenin levels does not always result in a change in expression of all Wnt target genes, which are also influenced by Bone morphogenetic protein (BMP) and Fibroblast growth factor (FGF) signaling 26, however, in the Apc2 model, increased incidence of nuclear β‐catenin translated into a small but significant upregulation of transcription levels of some Wnt target genes within the intestinal epithelia. Quantitative reverse transcriptase PCR (QRT‐PCR) using RNA extracted from isolated intestinal epithelium demonstrates subtle, yet significant over‐expression of Axin2, EphB3, and Cmyc, following the loss of Apc2, with no significant changes to Cyclin D1, EphB2, and Cd44 (Fig. 1C).

Thus, these data support previous publications which have shown that Apc2 is capable of regulating Wnt signaling, and indicates that the absence of Apc2 partially compromises the destruction of β‐catenin, resulting in an increase in total Wnt signaling within the intestinal epithelium.

Interestingly, the Wnt‐inhibitors *Dkk2*, *Groucho (GprK21)*, and *Wif1* are also significantly upregulated in response to Apc2 deficiency, indicating that the normal regulatory feedback loops are stimulated in response to the subtly elevated levels of Wnt signaling (Fig. 1D). As Wnt‐target gene expression levels are dependent on a number of different factors including levels of Wnt‐ligands, intracellular regulatory proteins such as those involved in the destruction complex as well as expression levels of Wnt‐inhibitors, there is not a direct relationship between levels of Wnt‐inhibitors levels and expression levels of Wnt‐target genes.

### Loss of Apc2 Results in Increased Apoptosis at the Base of the Intestinal Crypt

**Figure 2:**
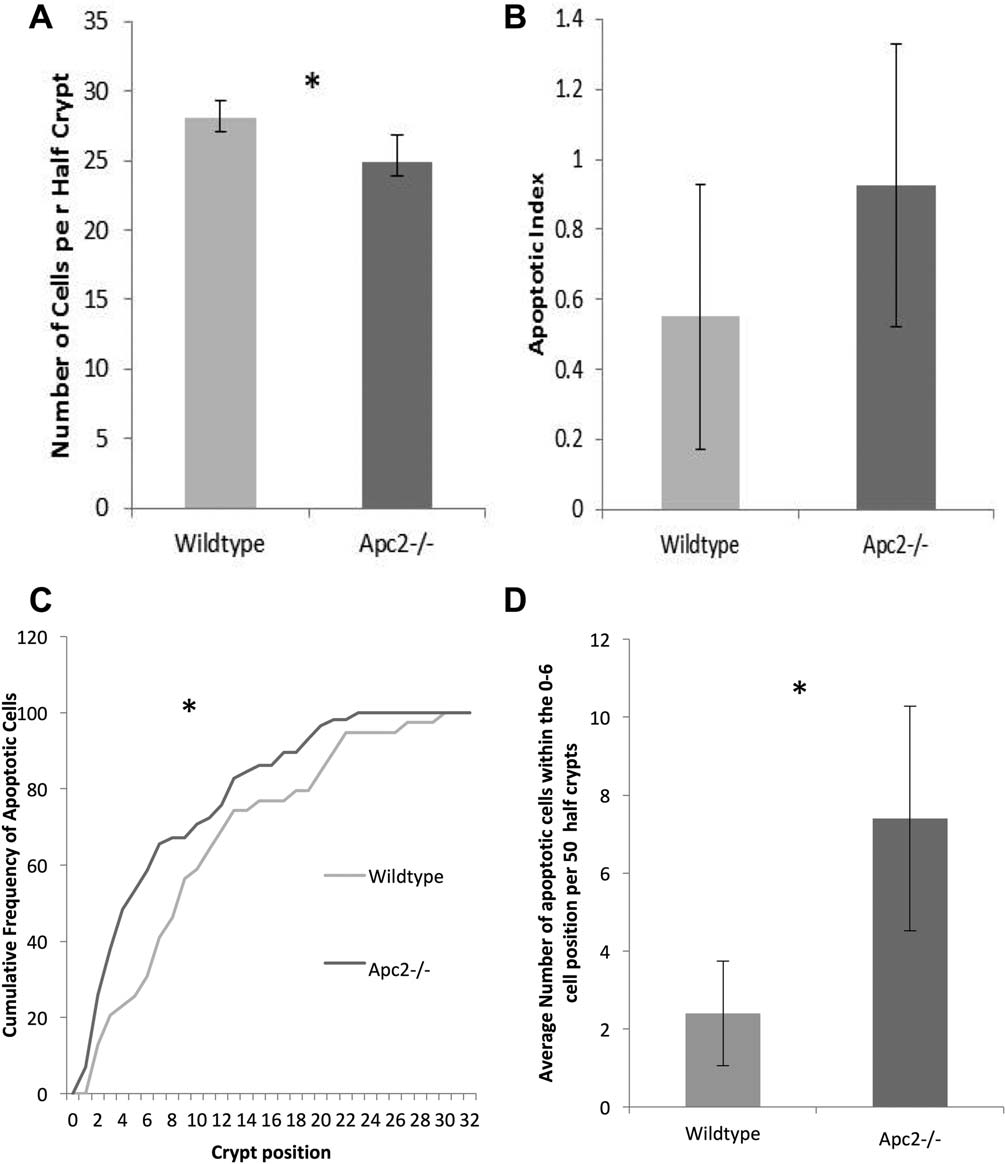
Intestinal homeostasis is regulated by Apc2. **(A):** Loss of Apc2 results in a decrease in number of cells per half‐crypt (*n* = 6). **(B):** Apc2 deficiency results in no significant change in number of apoptotic bodies as a percentage of crypt cells (*n* = 6). **(C):** The location of apoptotic bodies is significantly altered in absence of Apc2, with a higher proportion of them occurring at the base of the crypt. Asterisk denotes *p* value <.05. **(D):** Apc2 deficiency results in increased apoptosis at the base of the crypt in the 0–6 cell position (*n* = 6).

A detailed analysis of the intestines from the *Apc2^+/+^* intestine with *Apc2^−/−^* intestine from aged matched littermates was performed in order to assess the effect that subtly upregulated Wnt signaling has on intestinal homeostasis. Overall, the intestine showed no signs of gross abnormality in the absence of *Apc2* at 8 months old, with no change in numbers of the differentiated cell types, paneth cells, and goblet cells (Supporting Information Fig. 1). However, detailed analysis of the number of cells and location of apoptotic cells revealed subtle changes in intestinal homeostasis. Other than cell shedding which occurs at the tip of the villus, most apoptosis within the small intestine occurs at the crypt base 22. The total number of cells within the crypt was reduced in the *Apc2^−/−^* mice (Fig. 2A), and a trend for increased apoptosis within the crypt was observed (Fig. 2B). Although this increase was not significant, positional analysis of the apoptotic bodies indicated that while the location of apoptotic bodies in the intestinal epithelia of wildtype mice was comparable to the previously reported data with most of the apoptotic bodies within cell positions 0–10 22, apoptosis in the crypts of *Apc2^−/−^* mice occurred closer to the base. Furthermore, the level of apoptosis in the crypt base, positions 0–6, was significantly higher in the *Apc2^−/−^* (Fig. 2C, 2D). The crypt base positions 0–6 are thought to contain both of the proposed populations of ISCs; the crypt base columnar (CBC) cells interspersed between the paneth cells, and the +4 stem cell population. Thus, loss of *Apc2* results in a significant increase in cell death at the base of the intestinal crypt concomitant with the position of the ISCs and their niche, without impacting on the paneth cell number, implying increased apoptosis among the ISC populations. This trend for increased total apoptosis as a result of *Apc2* deficiency was replicated through the use of IHC for the apoptotic marker Caspase 3. A strong trend for increased apoptosis within the 0–6 cell position was also observed in the Caspase 3 IHC (Supporting Information Fig. 2).

Apc has a known role in the maintenance of genomic stability and loss of Apc can drive cell death through a number of mechanisms, not solely because of its role in regulating Wnt signaling. Due to the potential functional redundancy between the Apc proteins, DNA damage markers were assessed as a proxy for genomic instability within the small intestine. Apc2 deficiency had no apparent effect on levels of genomic instability, indicating that the increased cell death observed at the base of the crypt is not due to a potential role of Apc2 in regulating DNA damage (Supporting Information Fig. 3).

### *Apc2* Deficiency Results in Increased Levels of Expression of the Stem Cell Markers *Lgr5* and *Ascl2*, But Not an Increase in Stem Cell Number

**Figure 3:**
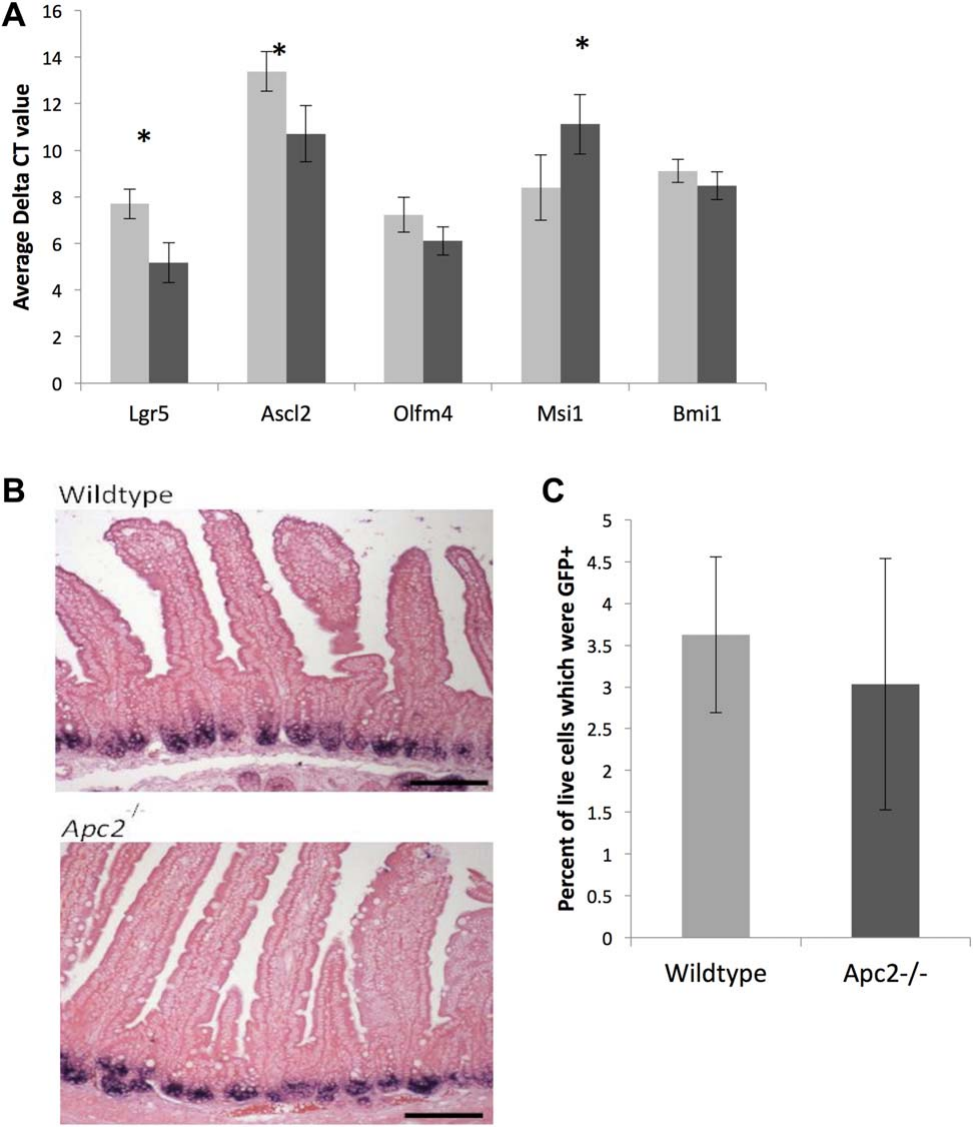
Apc2 regulation of the intestinal stem cell (ISC) markers. **(A):** Loss of Apc2 results in a change of expression levels of some ISC markers in the intestinal epithelium (*n* > 4). Light gray bars represent Wildtype tissue, dark gray bars represent Apc2^−/−^ intestinal tissue. Asterisk indicates a *p* value <.05. **(B):** Apc2 deficiency results in no mislocation of expression of Olfm4 mRNA as determined by in situ hybridization. **(C):** Fluorescence‐activated cell sorting (FACs) sorting of Lgr5+ cells reveal no significant change in number of Lgr5+ cells in Apc2^−/−^ intestinal epithelium (*n* = 4).

Controversy surrounding the utility of proposed genes as markers of the ISCs is further compounded by the proposition that the ISC compartment comprises of two distinct populations of cells; the rapidly cycling CBC cells responsible for homeostasis, and the quiescent “+4” population of cells thought to be responsible for intestinal repair after damage 27, 28. Because of this controversy, expression levels of proposed markers for both stem cell types were analyzed, with *Lgr5*, *Ascl2*, *Olfm4*, and *Msi1* expression representing the CBC population and *Bmi1* expression representing the +4 population. Interestingly, only *Lgr5* and *Ascl2* were significantly upregulated in response to loss of *Apc2* (Fig. 3), both of which are markers for the CBC cell population and recognized as Wnt target genes. However, the expression of the other markers associated with the CBC population were not elevated, and notably, *Msi1* expression levels were significantly downregulated in the *Apc2* deficient intestinal epithelium. *Msi1* is a Wnt target‐gene that was originally thought to be expressed by the CBC cell 29; however, it has now been shown that *Msi1* expression is not specifically limited to this compartment 30.

In situ hybridization analysis of the ISC marker *Olfm4* (described as co‐localizing with *Ascl2* 31) showed a normal expression pattern in the *Apc2* deficient intestine (Fig. 3B).

The *Lgr5Cre‐GFP* transgene created by Barker et al. enables the direct comparison of the number of Lgr5^+^ cells within the crypt through FACs sorting for GFP expression 25. Interestingly, despite an increase in expression of *Lgr5*, *Apc2* deficiency resulted no change in the percentage of intestinal epithelial cells of crypt origin which were *Lgr5Cre‐GFP* ^+^ when sorted for GFP (Fig. 3C). Thus, overall it appears that *Apc2^−/−^* mice survive and maintain intestinal homeostasis despite a potential aberration in ISCs, such that the higher levels of *Lgr5* expression does not correlate with an increase in Lgr5+ stem cell number. Furthermore, the increase of apoptosis at the base of the crypt and the reduced expression of *Msi1*, corroborate the notion of a potentially reduced ISC fitness.

### Loss of Apc2 Results in Compromised ISC Function

A potential caveat of the FACs analysis is associated with the known mosaicsm of the *Lgr5Cre‐GFP* transgene. It is also possible that the deficiency for *Apc2* results in a stronger silencing of the transgene rather than a reduction in the number of Lgr5 positive cells. To answer this conundrum, while also addressing the contradictory evidence between the levels of *Lgr5* expression and the number of *Lgr5* expressing cells, a stem cell functionality assay was utilized.

It has previously been shown that ISCs are capable of growing organoids in culture, and so organoid forming efficiency of disassociated intestinal crypts was used as a stem cell function assay. Disassociated intestinal crypts from wildtype and *Apc2^−/−^* mice were seeded at the same density, and the percentage of crypts which had formed organoids after 11 days post seeding was used as a measure of stem cell functionality. Crypts were used as an alternative to Lgr5^+^ single cells to avoid pre‐selection of an ISC population while ensuring a high enough organoid formation efficiency high enough to identify subtle changes.

**Figure 4:**
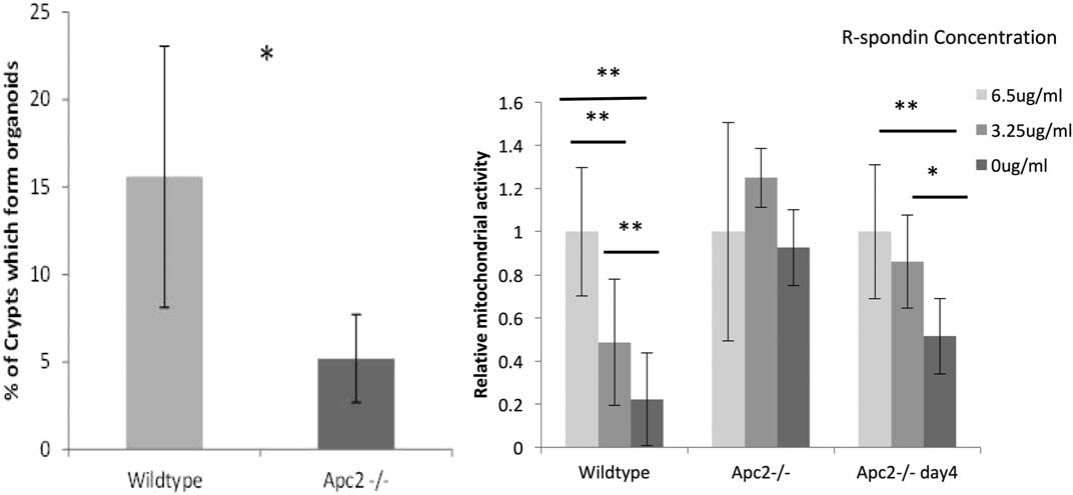
**(A):** Apc2 deficiency significantly reduces organoid formation efficiency of intestinal crypts *n* ≥ 5 (Wildtype 15.6% ± 7.5% vs. Apc2^−/−^ 5.2% ± 2.5%, two‐tailed *t* test, *p* = .014). **(B):** Mitochondrial activity of Apc2^−/−^ organoids is higher than their wildtype counterparts after 3 and 4 days of culture with reduced R‐spondin availability as assessed using PrestoBlue viability assay. * indicates *p* value of <.05. ** indicates *p* value of <.01.

Interestingly, an increase in expression of *Lgr5* was associated with a decrease in ISC functionality, with many fewer *Apc2^−/−^* crypts capable of forming organoids than their wildtype counterparts, which is in‐line with the observed increase of apoptosis at the base of the crypt (Fig. 4A).

**Figure 5:**
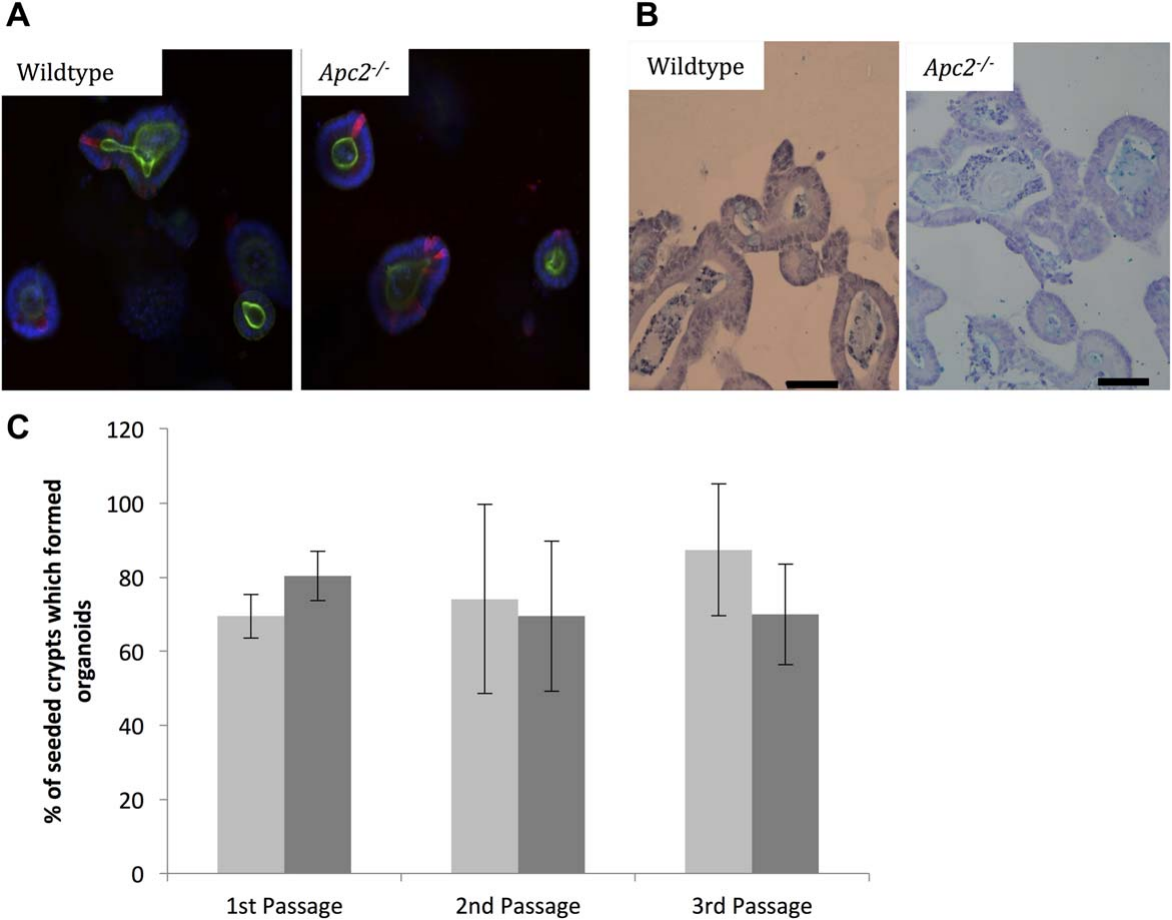
**(A):** Wholemount Immunofluorescence (IF) of intestinal organoids reveal no overt changes in number of paneth cells as a result of Apc2 deficiency, Red = Lysozyme, Green= phalloidin. **(B):** Immunohistochemistry for Alcian blue shows no overt change in number of goblet cells as a result of Apc2 deficiency. Scale bars represent 50 μm. **(C):** Passage efficiency was unchanged as a result of Apc2 deficiency, Light gray bars represent wildtype intestinal organoids and dark gray bars represent Apc2^−/−^ intestinal organoids, *n* > 10 wells.

Of the organoids that did grow, however, there was no difference in the growth rate (Supporting Information Fig. 4) or size of the organoids, indicating that the organoids which did grow were normal. IHC on formalin fixed, paraffin embedded organoids, and immunofluorescence on wholemount organoids revealed that *Apc2* deficient organoids are also capable of developing differentiated cell types such as goblet and paneth cells (Fig. 5A, 5B). This is supported by an alternative stem cell function assay, the self‐renewal assay. In this assay, wildtype and *Apc2^−/−^* organoids were grown, and the passage efficiency was measured. Both cultures were capable of being passaged more than three times and there were no differences in the self‐renewal efficiencies between cultures (Fig. 5C). As organoid cultures have been shown to be genomically stable, we would not anticipate this passage efficiency to alter longer‐term 32, although this was not explored.

Wildtype organoids cultures are only capable of growth in the presence of R‐spondin within the culture medium for cell survival and organoid growth, whereas organoids grown from Wnt‐activated stem cells such as those derived from *Apc^flox/flox^* mice are not 33. In order to assess the levels of Wnt activation within the ISC compartment as a result of *Apc2* deficiency, intestinal crypts from both wildtype and *Apc2^−/−^* mice were cultured in 6.5 μg/ml, 3.25 μg/ml, and 0 μg/ml R‐spondin. Wildtype crypts die in culture in the absence of R‐spondin by day 2–3, but interestingly *Apc2^−/−^* organoids survived until day 3 and only began to die by day 4 (Fig. 4B). In order to quantify organoid survival at varying concentrations of R‐spondin within the media, the Prestoblue assay, a mitochondrial activity assay was used. As day 3 appeared to be the critical day for survival, mitochondrial activity was assessed on days 3 and 4 post seeding 34. This assay showed that compared to wildtype, mitochondrial activity of *Apc2^−/−^* organoids is still high in the absence of R‐spondin at day 3, but has dropped to wildtype levels by day 4. This indicates that despite an apparent decrease in the number of functional stem cells (cells capable of forming organoids in culture), the ISCs which have overcome the apoptotic effect of increased Wnt signaling are less dependent of R‐spondin, indicating that they are more highly Wnt‐activated than wildtype.

## Discussion

It is widely accepted that Wnt signaling levels are tightly regulated within the intestine in order to maintain homeostasis, with changes in Wnt signaling having potentially disastrous effects on the structural integrity of the tissue. Although the relationship between Wnt signaling and the maintenance of the ISC population has been investigated when Wnt‐signaling levels are grossly altered, the effect of subtly altered Wnt signaling on the ISC compartment has so far remained unexplored.

A constitutive Apc2 knockout proved to be a suitable model to represent subtly altered levels of Wnt signaling within the intestine. In order to analyze the effect of these altered Wnt levels on the ISC compartment, a wide range of techniques were used, which showed that loss of *Apc2* resulted in an increased expression of the ISC markers *Lgr5* and *Ascl2* but no change in the percentage of *Lgr5^+^* cells. Functionality of the ISCs was explored through the use of organoid culture techniques, and showed that loss of *Apc2* leaves intestinal crypts less efficient at forming organoids in culture, a capability which is associated with functionality of the ISC compartment. Of the *Apc2^−/−^* organoids which did grow however there was no difference in self‐renewal efficiency compared to wildtype. These observations lead to the hypothesis that within an *Apc2^−/−^* murine intestine there is a heterogeneous population of ISCs, a proportion of which can behave normally and form organoids which grow in the same manner to wildtype organoids, and others which are more highly Wnt activated are more likely to die, (as observed by an increase in apoptosis within the first four cell positions within the crypt) and are less fit as ISCs (as observed by the overall reduced intestinal organoid formation efficiency).

Given that the levels of ISC markers did not correlate with intestinal organoid formation efficiency, it must be assumed that either levels of expression of these markers do not directly impact ISC fitness, or that organoid formation is a poor readout of ISC activity levels. The one marker which did show a correlation was expression of *Msi1*. Recently, it has been proposed that Msi protein levels, although not a good indication of the CBC population, play an important role in the activation of the quiescent ISC population into the cell cycle in response to injury 35. Intestinal organoid formation as described here is essentially an assay of damage response after crypt isolation. The subtle Wnt deregulation caused by *Apc2* loss, and the associated reduced *Msi1* expression, may well impact the ability of intestinal crypts to activate their quiescent stem cell population in response to trauma. This could explain why intestinal homeostasis was largely unaffected by Apc2 deficiency, but had an impact on ISC fitness.

## Conclusion

This hypothesis is supported by the idea that levels of Wnt signaling within the intestinal epithelium must be tightly regulated 36. The Wnt “just‐right” hypothesis presents the idea that changes in Wnt signaling can affect ISC survival. In a number of tumor models, it has been shown that an increase in Wnt signaling can result in an increase in apoptosis, thereby reducing the tumor burden 37. Thus, we show for the first time that even subtle misregulation of Wnt signaling can impact on the function and fitness of the ISC population.

## Author Contributions

M.A.Y.: designed the research, managed the mouse intercrosses, performed organoid culture and associated imaging, performed IHC, performed qRT‐PCR, analyzed data, manuscript drafting; C.S.D.: designed the research, performed IHC; K.R.R.: designed the research; A.R.C.: designed the research; E.T.: managed the mouse intercrosses; R.J. performed qRT‐PCR, performed western blotting; All authors: critically reviewed the manuscript, read and approved the final manuscript. The now late Professor Alan Clarke was aware of the results of the study and had approved of the manuscript plan.

## Disclosure of Potential Conflicts of Interest

The authors indicated no potential conflicts of interest.

## Note Added in Proof

This article was published online on 13 October 2017. Minor edits have been made that do not affect data. This notice is included in the online and print versions to indicate that both have been corrected 29 December 2017.

## Supporting information

Supplementary Figures
