# Supplementary material for: Subtle Deregulation of the Wnt‐Signaling Pathway Through Loss of Apc2 Reduces the Fitness of Intestinal Stem Cells
Source: Stem Cells. 2017 Oct 13;36(1):114–22. doi: 10.1002/stem.2712 (PMC5765519; doi:10.1002/stem.2712)
Supplement: Supplementary file 1 — Supplementary Figures [file STEM-36-114-s001.pptx]

## Slide 1
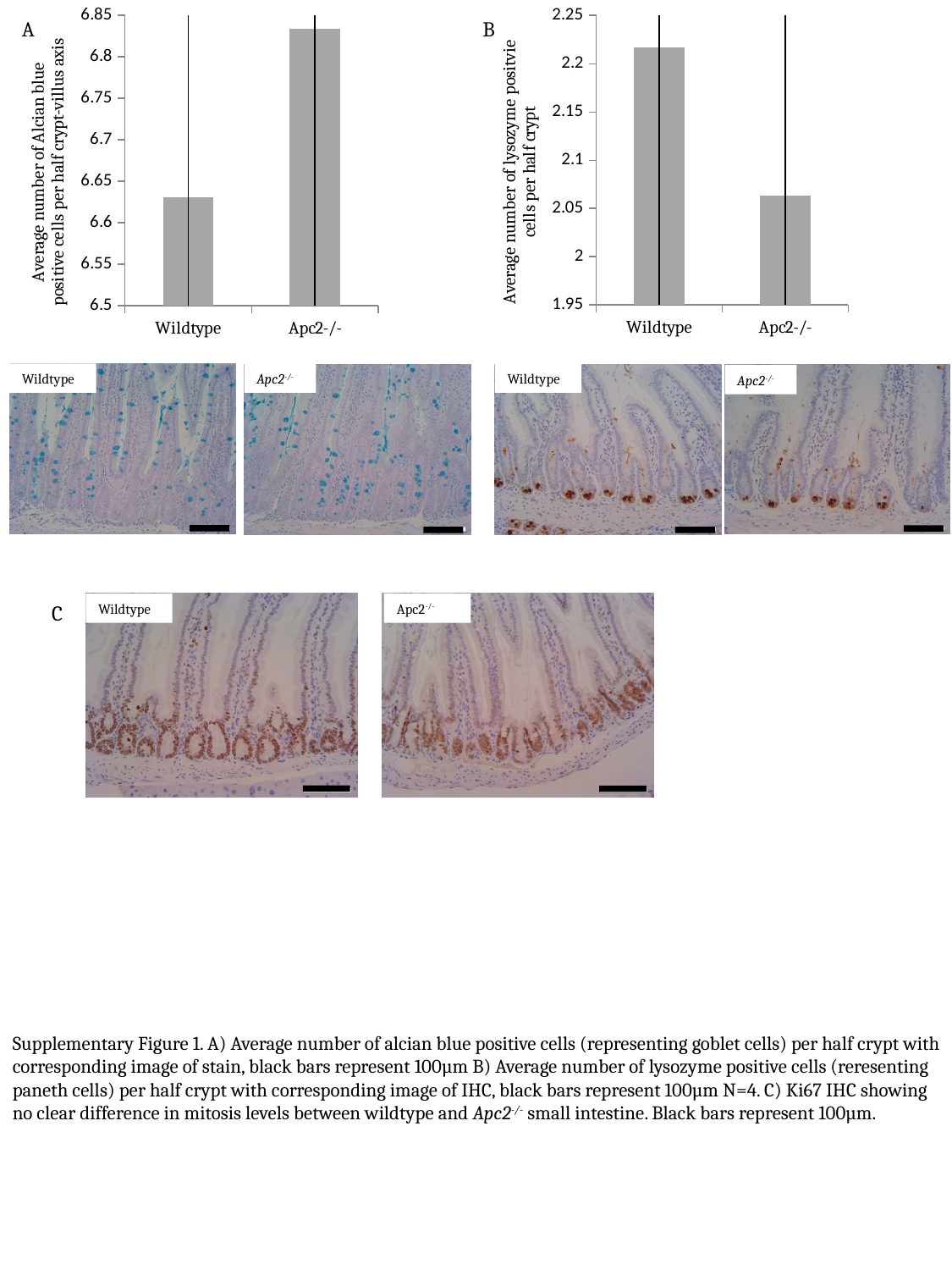

### Chart
| Category | |
|---|---|
| Wildtype | 2.216666666666667 |
| Apc2-/- | 2.063333333333333 |
### Chart
| Category | |
|---|---|
| Wildtype | 6.630952380952381 |
| Apc2-/- | 6.833333333333333 |A
B
Wildtype
Apc2-/-
Wildtype
Apc2-/-
C
Wildtype
Apc2-/-
Supplementary Figure 1. A) Average number of alcian blue positive cells (representing goblet cells) per half crypt with corresponding image of stain, black bars represent 100µm B) Average number of lysozyme positive cells (reresenting paneth cells) per half crypt with corresponding image of IHC, black bars represent 100µm N=4. C) Ki67 IHC showing no clear difference in mitosis levels between wildtype and Apc2-/- small intestine. Black bars represent 100µm.

## Slide 2
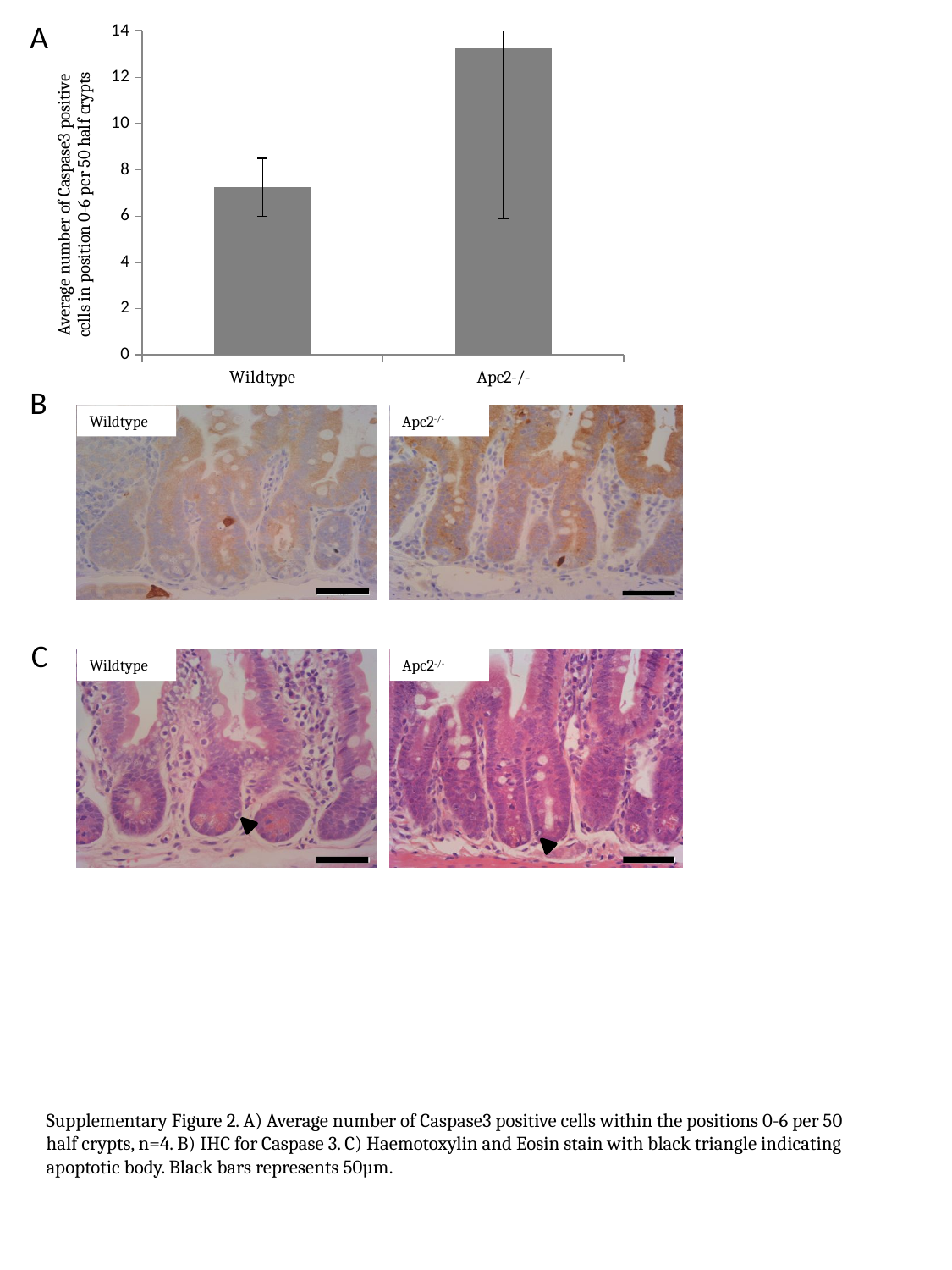

A
### Chart
| Category | |
|---|---|
| Wildtype | 7.25 |
| Apc2-/- | 13.25 |B
Apc2-/-
Wildtype
C
Wildtype
Apc2-/-
Supplementary Figure 2. A) Average number of Caspase3 positive cells within the positions 0-6 per 50 half crypts, n=4. B) IHC for Caspase 3. C) Haemotoxylin and Eosin stain with black triangle indicating apoptotic body. Black bars represents 50µm.

## Slide 3
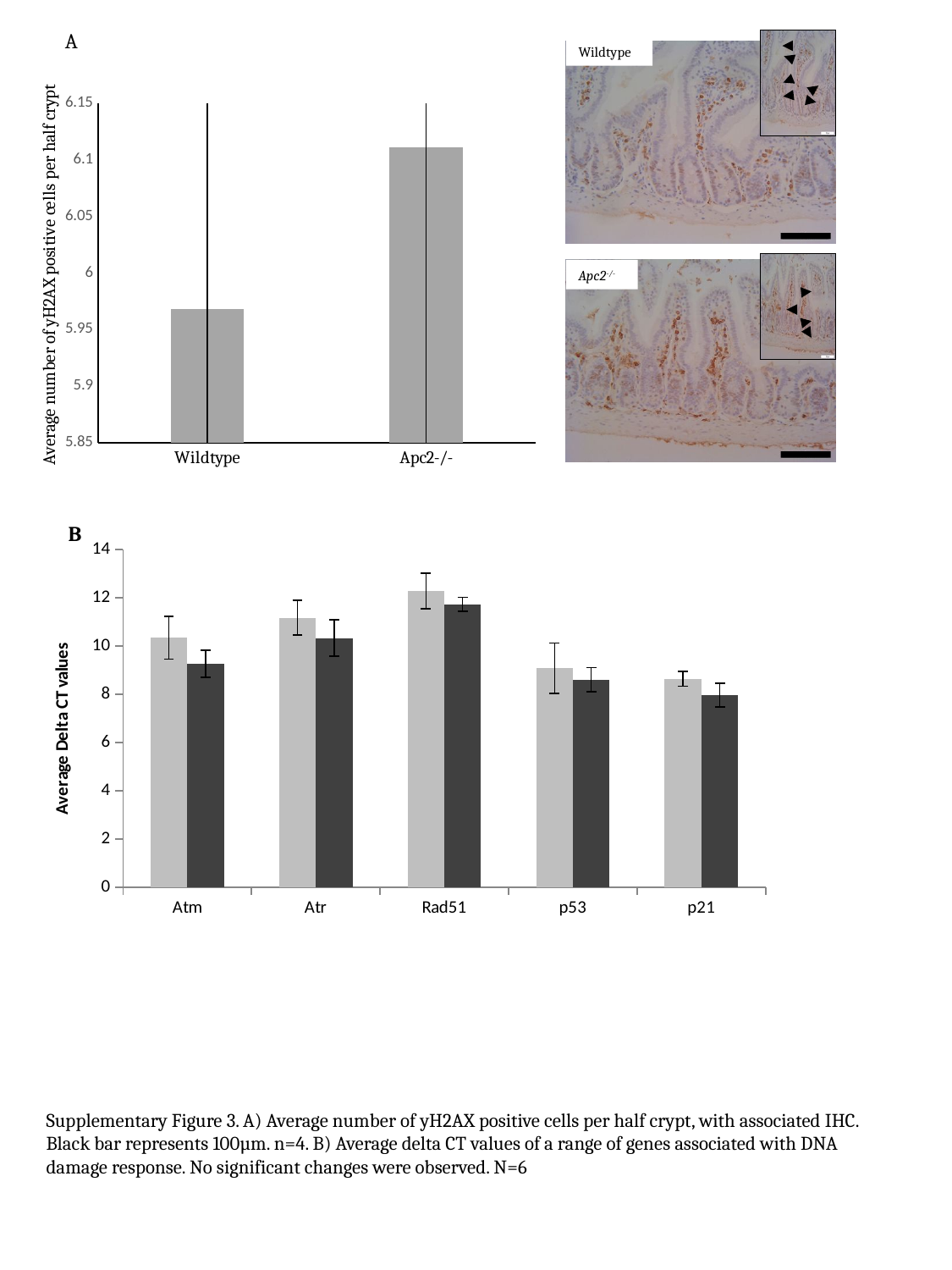

A
### Chart
| Category | |
|---|---|
| Wildtype | 5.968000000000001 |
| Apc2-/- | 6.111428571428569 |
Wildtype
Apc2-/-
B
### Chart
| Category | WT | Apc2-/- |
|---|---|---|
| Atm | 10.3446023464203 | 9.259608268737795 |
| Atr | 11.16679692268372 | 10.33037710189819 |
| Rad51 | 12.27771306037903 | 11.71997833251953 |
| p53 | 9.085870504379272 | 8.60384225845337 |
| p21 | 8.643187284469604 | 7.971553802490237 |Supplementary Figure 3. A) Average number of yH2AX positive cells per half crypt, with associated IHC. Black bar represents 100µm. n=4. B) Average delta CT values of a range of genes associated with DNA damage response. No significant changes were observed. N=6

## Slide 4
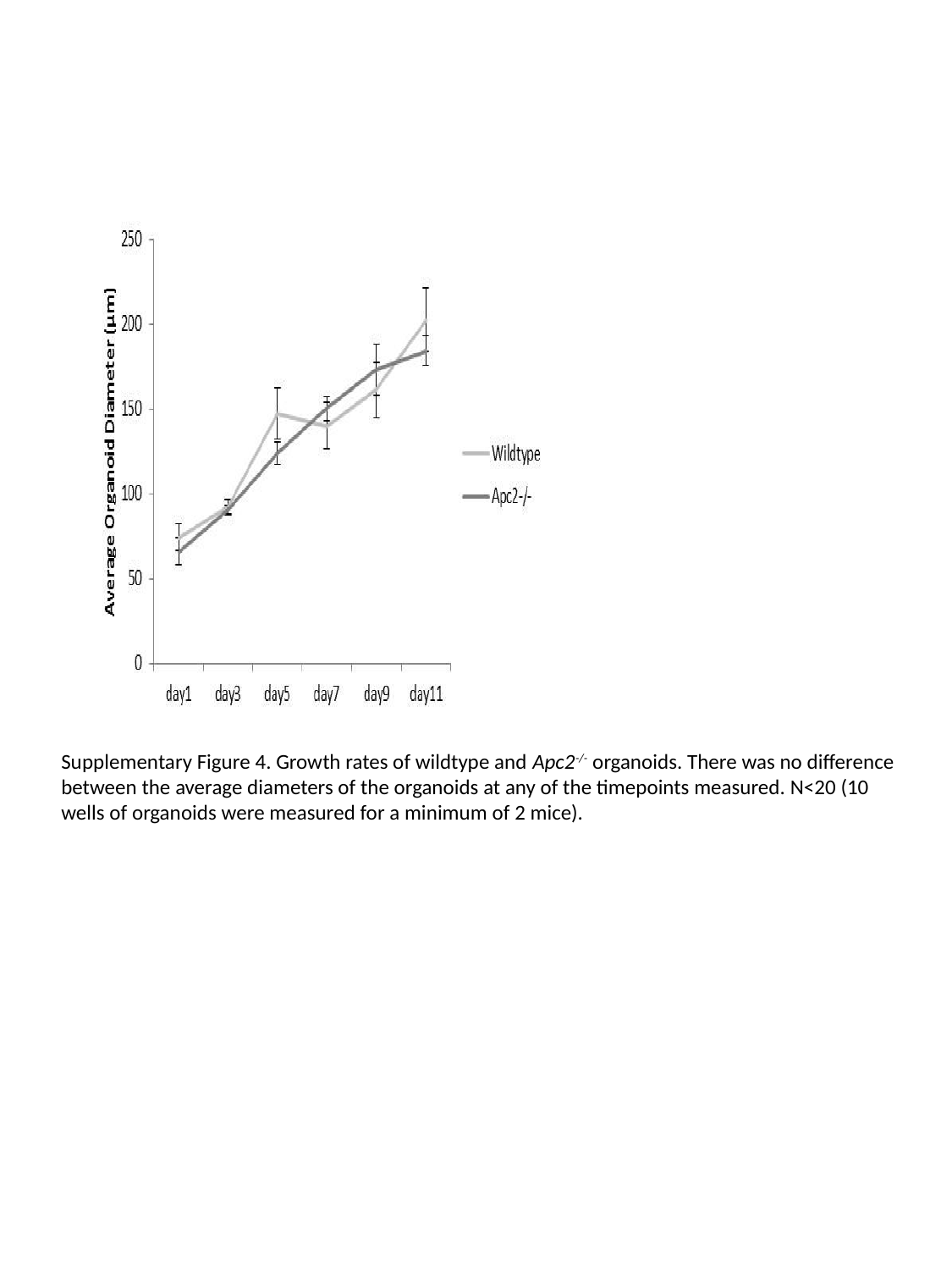

Supplementary Figure 4. Growth rates of wildtype and Apc2-/- organoids. There was no difference between the average diameters of the organoids at any of the timepoints measured. N<20 (10 wells of organoids were measured for a minimum of 2 mice).

## Slide 5
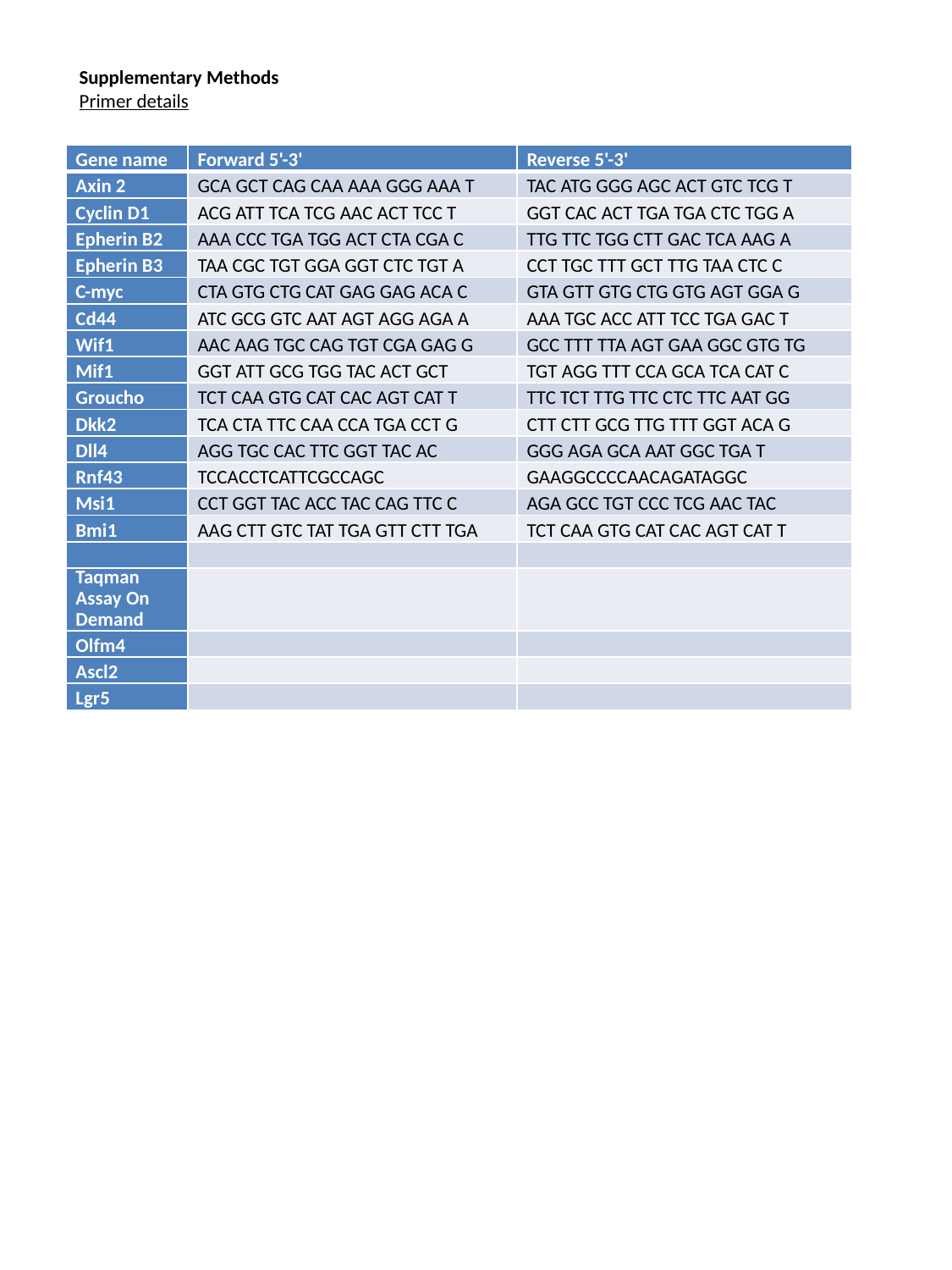

Supplementary Methods
Primer details
| Gene name | Forward 5'-3' | Reverse 5'-3' |
| --- | --- | --- |
| Axin 2 | GCA GCT CAG CAA AAA GGG AAA T | TAC ATG GGG AGC ACT GTC TCG T |
| Cyclin D1 | ACG ATT TCA TCG AAC ACT TCC T | GGT CAC ACT TGA TGA CTC TGG A |
| Epherin B2 | AAA CCC TGA TGG ACT CTA CGA C | TTG TTC TGG CTT GAC TCA AAG A |
| Epherin B3 | TAA CGC TGT GGA GGT CTC TGT A | CCT TGC TTT GCT TTG TAA CTC C |
| C-myc | CTA GTG CTG CAT GAG GAG ACA C | GTA GTT GTG CTG GTG AGT GGA G |
| Cd44 | ATC GCG GTC AAT AGT AGG AGA A | AAA TGC ACC ATT TCC TGA GAC T |
| Wif1 | AAC AAG TGC CAG TGT CGA GAG G | GCC TTT TTA AGT GAA GGC GTG TG |
| Mif1 | GGT ATT GCG TGG TAC ACT GCT | TGT AGG TTT CCA GCA TCA CAT C |
| Groucho | TCT CAA GTG CAT CAC AGT CAT T | TTC TCT TTG TTC CTC TTC AAT GG |
| Dkk2 | TCA CTA TTC CAA CCA TGA CCT G | CTT CTT GCG TTG TTT GGT ACA G |
| Dll4 | AGG TGC CAC TTC GGT TAC AC | GGG AGA GCA AAT GGC TGA T |
| Rnf43 | TCCACCTCATTCGCCAGC | GAAGGCCCCAACAGATAGGC |
| Msi1 | CCT GGT TAC ACC TAC CAG TTC C | AGA GCC TGT CCC TCG AAC TAC |
| Bmi1 | AAG CTT GTC TAT TGA GTT CTT TGA | TCT CAA GTG CAT CAC AGT CAT T |
| | | |
| Taqman Assay On Demand | | |
| Olfm4 | | |
| Ascl2 | | |
| Lgr5 | | |

## Slide 6
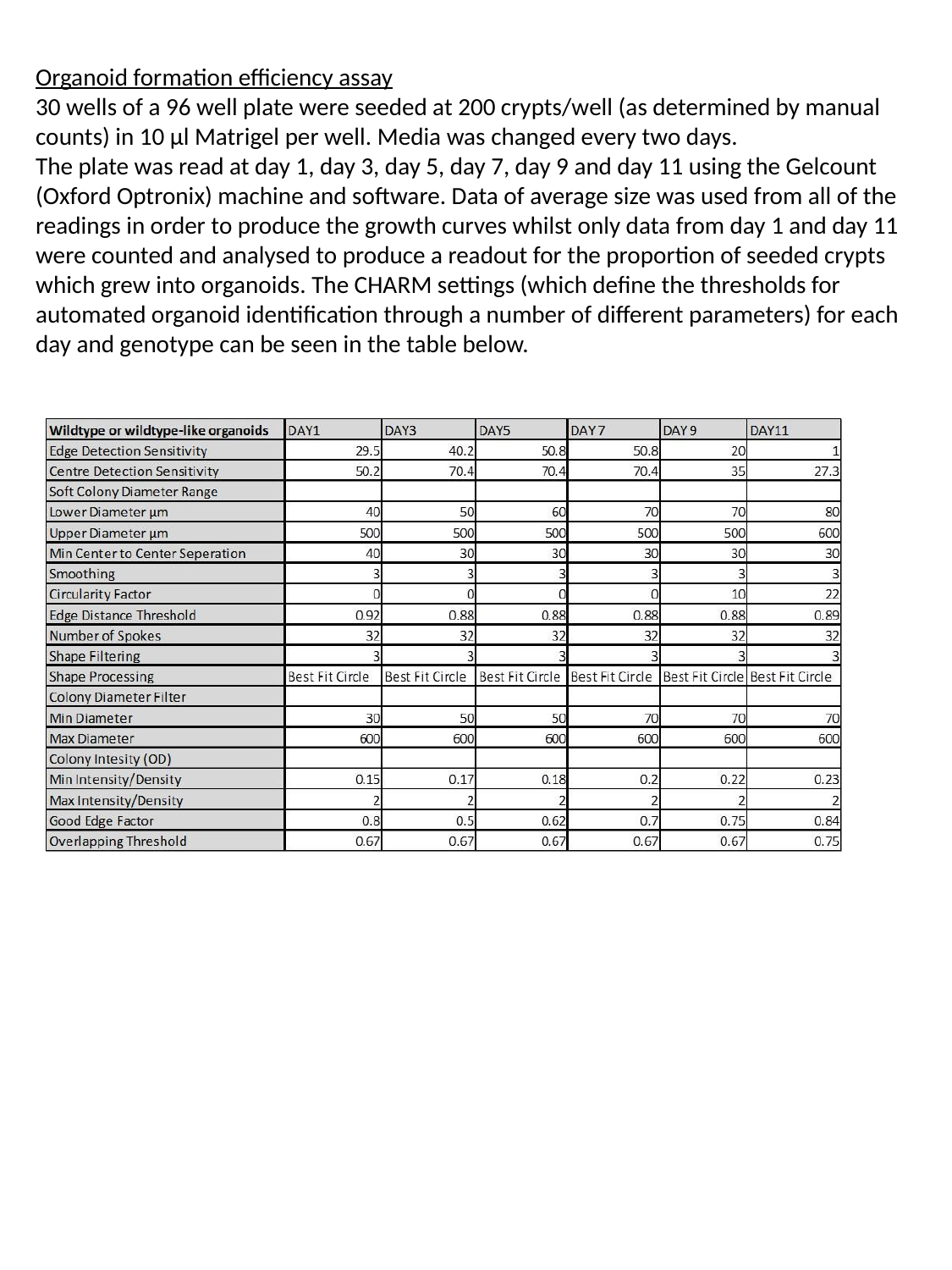

Organoid formation efficiency assay
30 wells of a 96 well plate were seeded at 200 crypts/well (as determined by manual counts) in 10 μl Matrigel per well. Media was changed every two days.
The plate was read at day 1, day 3, day 5, day 7, day 9 and day 11 using the Gelcount (Oxford Optronix) machine and software. Data of average size was used from all of the readings in order to produce the growth curves whilst only data from day 1 and day 11 were counted and analysed to produce a readout for the proportion of seeded crypts which grew into organoids. The CHARM settings (which define the thresholds for automated organoid identification through a number of different parameters) for each day and genotype can be seen in the table below.
